# Anticancer Potential of Indole Phytoalexins and Their Analogues

**DOI:** 10.3390/molecules29102388

**Published:** 2024-05-19

**Authors:** Martina Zigová, Radka Michalková, Ján Mojžiš

**Affiliations:** Department of Pharmacology, Faculty of Medicine, Pavol Jozef Šafárik University, 040 01 Košice, Slovakia; chripkova.martina@gmail.com

**Keywords:** indole phytoalexins, cancer, brassinin, camalexin, antiproliferative

## Abstract

Indole phytoalexins, found in economically significant *Cruciferae* family plants, are synthesized in response to pathogen attacks or stress, serving as crucial components of plant defense mechanisms against bacterial and fungal infections. Furthermore, recent research indicates that these compounds hold promise for improving human health, particularly in terms of potential anticancer effects that have been observed in various studies. Since our last comprehensive overview in 2016 focusing on the antiproliferative effects of these substances, brassinin and camalexin have been the most extensively studied. This review analyses the multifaceted pharmacological effects of brassinin and camalexin, highlighting their anticancer potential. In this article, we also provide an overview of the antiproliferative activity of new synthetic analogs of indole phytoalexins, which were synthesized and tested at our university with the aim of enhancing efficacy compared to the parent compound.

## 1. Introduction

**Figure 1:**
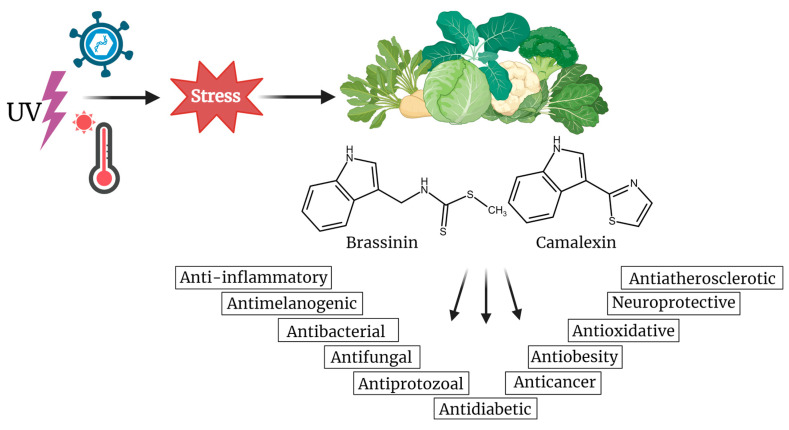
Biological effects of indole phytoalexins, brassinin and camalexin, produced in plants of the *Cruciferae* (*Brassicaceae*) family under stress conditions. Created with BioRender.com.

Cancer remains one of the most formidable challenges to human health, with its incidence continuing to rise globally [1]. Despite advancements in treatment modalities, the quest for novel and more effective anticancer agents persists. In recent years, natural compounds have garnered considerable attention due to their diverse pharmacological activities and potential as leads for drug development [2,3,4]. Among these, indole phytoalexins have emerged as intriguing candidates for exploration due to their diverse biological activities and potential as anticancer agents. Indole phytoalexins, produced by plants of the *Cruciferae* (*Brassicaceae*) family, are natural compounds formed in response to diverse stresses, including pathogen attacks. These low-molecular-weight antimicrobial secondary metabolites are generally synthesized de novo by plants facing various stressors like biological factors (bacteria, fungi, viruses), physical stressors (UV radiation, heat shock, injury), and chemical stressors (heavy metals) [5]. Their notability stems from the considerable attention they have garnered due to potential health benefits, particularly their recognized anticancer properties (Figure 1). Moreover, they display a broad spectrum of antifungal effects, antitrypanosomal properties [6], and modest antibacterial activity [7,8]. In addition, they have been found to have an antiaggregatory effect on the cerebrospinal fluid in multiple sclerosis patients [9]. Moreover, indole phytoalexins possess cancer chemopreventive attributes [10] and exhibit antiproliferative activity on several human cancer cell lines [11,12,13,14,15,16,17].

Our review, published in 2016, emphasized the essential characteristics of indole phytoalexins, with a specific focus on their significant antiproliferative effects [18]. Over the subsequent years, we have undertaken the synthesis and evaluation of numerous novel derivatives of indole phytoalexins, propelled by their outstanding antiproliferative characteristics. This article aims to offer an updated review encompassing the latest advancements, building upon our previous comprehensive analysis.

## 2. Origin and Structural Diversity of Indole Phytoalexins

An indole-based group of phytoalexins has been isolated from economically and nutritionally important vegetables, including cabbages, turnips, rapeseed, canola, Brussels sprouts, cauliflower, and others [19]. In addition to indole phytoalexins, plants from the Brassicaceae family are capable of producing various other sulfur-containing secondary metabolites known as glucosinolates. Interestingly, there is a strong likelihood that glucosinolates and indole phytoalexins, traditionally viewed as distinct compound groups, share a common evolutionary origin and are connected at the biosynthetic level. Indole glucosinolates are plausible direct precursors to a subset of indole phytoalexins, emphasizing their mutual biosynthetic pathway. Several labeling studies have demonstrated the de novo biosynthesis of indole phytoalexins from glucosinolates via isothiocyanates [20,21,22,23]. Furthermore, even in the case of camalexin biosynthesis, which is mostly independent of the indole glucosinolate pathway, a similar biochemical mechanism involving at least two identical enzymes is utilized to incorporate sulfur into the molecular structure. This evidence suggests a common underlying mechanism for sulfur integration in diverse phytoalexins within the *Brassicaceae* family. Unlike glucosinolates, which are present constitutively, indole phytoalexins are synthesized only in response to pathogen attack. This is a characteristic of the plant’s innate immune response, involving the transcriptional regulation of biosynthetic genes [24].

Since 1986, a total of 54 indole phytoalexins have been investigated and characterized chemically. The chemical structures of all cruciferous phytoalexins are illustrated in the review authored by Pedras and Abdoli in 2017 [25]. The basic structure of indole phytoalexins is an indole, oxindole, or indoline nucleus with a linear chain or annexed heterocycle. Certain indole phytoalexins exhibit distinct structural characteristics, including a thiazoline ring attached in a spiro arrangement. Most of the phytoalexins found in cruciferous plants have been identified as originating from (*S*)-tryptophan [21,25,26,27,28]. Subsequently, a new structural group derived from (*S*)-phenylalanine was discovered [29,30]. Notably, watercress is the sole crucifer species known to synthesize phytoalexins through two distinct pathways, originating from the primary precursors amino acids (*S*)-tryptophan and (*S*)-phenylalanine [26,27]. Among the indole phytoalexins derived from (*S*)-phenylalanine are nasturlexins and tridentatol C, which were isolated from winter cress and upland cress. Included in the category of nitrile-containing phytoalexins are tenualexin, indolyl-3-acetonitrile, caulilexin C, and arvelexin. Tenualexin is probably synthesized through the biosynthesis of corresponding indolyl acetaldoximes, while cyclonasturlexin is derived biosynthetically from brassinin through the indolyl glucosinolate glucobrassicin pathway [28]. Given the extensive number of crucifer species (exceeding 3700 species) and the relatively small number of species (<50) studied for phytoalexin production, it is probable that phytoalexin structures originating from phenylalanine/tyrosine and their homologs will exhibit a variety of functional groups similar to those derived from (*S*)-tryptophan. Isocyalexin A was the first isocyanide (or isonitrile, –NC) documented in the plant kingdom, despite the prevalence of cyanide (or nitriles, –CN)-containing metabolites in plants [25]. Brassinin, 1-methoxybrassinin, (*S*)-(−)-spirobrassinin, (*R*)-(+)-1-methoxyspirobrassinin, 1-methoxyspirobrassinol, *trans*-(2*R*,3*R*)-(−)-1-methoxyspirobrassinol methyl ether, cyclobrassinin, sinalbin B, and rutalexin are typical representatives of naturally occurring cruciferous phytoalexins of dithiocarbamate, spiroindoline[3,5′]thiazolidine, and thiazino[6,5-b]indole type. Camalexin, despite the shared indole core and presence of a sulfur atom in its structure, seems to constitute a separate structural subgroup among other *Brassicaceae* phytoalexins [5,20].

These indole compounds, isolated from various cabbage plants, exhibit intriguing pharmacological characteristics that deserve attention in the context of cancer research. These substances are characterized by significant antiproliferative effects, which we described in our previous review [18]. Since our last comprehensive overview, brassinin and camalexin have been the most extensively studied. 

## 3. Brassinin

Brassinin is one of the first isolated indole phytoalexins ever, and it was extracted from Chinese cabbage after infection by the bacterium *Pseudomonas cichorii* [31]. It is suggested that more than 30 cruciferous phytoalexins originate from brassinin or its analogs [20,32]. This natural substance is recognized for its diverse pharmacological properties, including antibacterial, antifungal, antiobesity, anti-inflammatory, antimelanogenic, antidiabetic, antiatherosclerotic, and anticancer effects [7,8,33,34,35,36,37,38].

### 3.1. Anticancer Potential of Brassinin

Brassinin demonstrates anticancer potential in various cancers such as myelogenous leukemia [39], colon [40], prostate [41], breast [42], lung [43], and nasopharyngeal [44], hepatocellular carcinoma [17]. It shows great potential as a prospective anticancer agent due to its ability to induce apoptosis, inhibit angiogenesis, and modulate crucial signaling pathways involved in cancer development and progression, such as the NF-κB pathway, Janus kinase/signal transducer and activator of transcription 3 (JAK-STAT3), pathway and Phosphoinositide 3-kinase/protein kinase B/mammalian target of rapamycin (PI3K/Akt/mTOR) pathway. By interfering with these pathways, brassinin potentially impedes the survival and proliferation of cancer cells. The results of the study by Park et al. (2021) suggest that brassinin initiates apoptosis by inducing the expression of p53 and p21, depending on the dosage and duration of exposure. Interestingly, brassinin induces apoptosis in colon cancer cells with wild-type p53 (HCT116p53+/+) in a dose-dependent manner, while having no such effect on cancer cells lacking functional p53 (null-type). In addition to apoptosis, p53 activation promotes ferroptosis, senescence, and cell cycle arrest, while inhibiting migration and metastasis in cancer cells. Moreover, brassinin dose-dependently inhibits the expression of subunit of the Carbon catabolite repression 4–negative on TATA-less (CCR4-NOT) complex (CNOT2) in these cells [38]. CNOT2 acts as an oncogene in various types of cancer cells, contributing to processes such as apoptosis, metastasis, angiogenesis, and autophagy [45,46,47].

**Figure 2:**
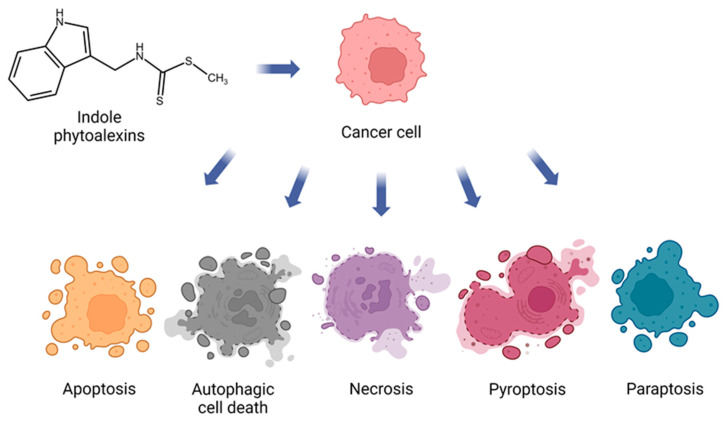
Indole phytoalexins induce tumor cell death by different mechanisms. Created with BioRender.com.

Generally, indole phytoalexins can induce cancer cell death by various mechanisms (Figure 2).

**Figure 3:**
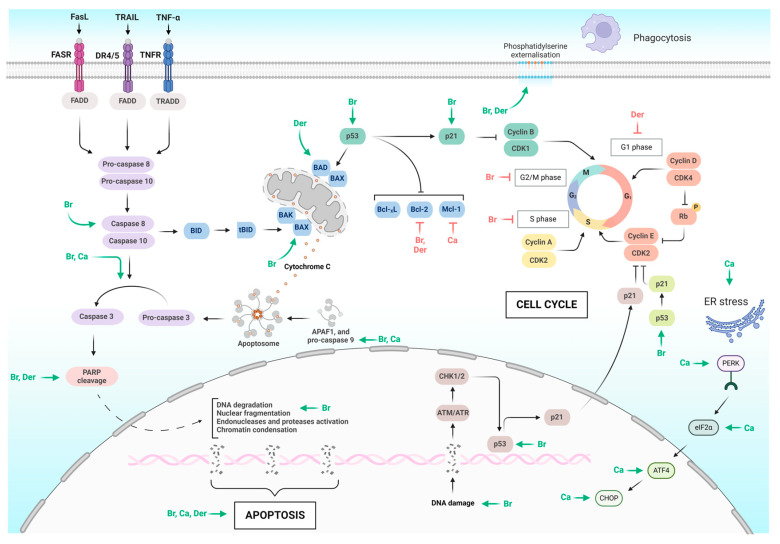
Induction of apoptosis and cell cycle blockade by indole phytoalexins and their derivatives. Green arrow—induction, red T bar—inhibition, Br—brassinin, Ca—camalexin, Der—derivatives. Created with BioRender.com.

Among them, brassinin induces three distinct forms of cell death—apoptosis, autophagy, and paraptosis—in chronic myelogenous leukemia (CML) cells through the activation of the mitogen-activated protein kinase (MAPK) signaling pathway [39]. The crucial role of the MAPK pathway in regulating both apoptosis (Figure 3) and autophagy has been widely acknowledged for an extended period [48,49]. In addition to activating this pathway, brassinin enhances the phosphorylation of c-Jun N-terminal kinase (JNK), extracellular signal-regulated kinase (ERK), and p38 in leukemia cells [39]. The activation of p-JNK and p-ERK within the MAPK pathway has been identified as crucial for paraptosis, while the p38 pathway in the MAPK pathway contributes to the formation of paraptosis vacuoles [50,51]. The results of this study indicated that paraptosis induced by brassinin was accompanied by increased production of ROS, mitochondrial damage, endoplasmic reticulum (ER) stress, and cytoplasmic vacuolation. Moreover, brassinin disrupted the glutathione/oxidized glutathione ratio (GSH/GSSG) balance and elevated the levels of ER stress-related proteins such as activating transcription factor (ATF4) and CCAAT/enhancer-binding protein homologous protein (CHOP). Another sign that brassinin induced paraptosis was its reduction in Alix expression. The influence of brassinin on autophagy activation was affirmed by the expression of the microtubule-associated protein 1A/1B-light chain 3 (LC3) and an acridine orange assay [39]. LC3 serves as a reliable marker for monitoring autophagy activity, indicating its presence during active autophagy in the cell [52]. In terms of inducing apoptosis in CML cells, brassinin activates caspase-8, caspase-9, and caspase-3, leading to PARP cleavage and an increase in the distribution of cells in the sub-G1 phase [39]. The induction of apoptosis by brassinin, through increased activity of caspase-3 and caspase-9, was observed in nasopharyngeal cancer cells (C666-1). Additionally, it elevated the expression of Bax and reduced Bcl-2. Flow cytometry analysis demonstrated that brassinin administration enhanced the G0/G1 ratio and decreased the proportion of cells in the ‘S’ and ‘G2/M’ phases in C666-1 cells [44]. The study by Kwon et al. (2023) also demonstrated the pro-apoptotic effects of brassinin, specifically on prostate cancer cells (PC-3, DU145, LNCaP). This research suggested that androgen-independent PC-3 cells exhibited higher susceptibility to brassinin compared to DU145 and androgen-dependent LNCaP cells while showing minimal impact on normal prostatic epithelial (RWPE-1) cells. Brassinin induced apoptosis in PC-3 cells by downregulating pro-PARP, pro-caspase 3, and Bcl2, indicating a caspase-dependent apoptotic effect. Notably, brassinin displayed potential in inducing apoptosis in PC-3 cells by increasing the sub-G1 population and TUNEL-positive cells. Furthermore, it demonstrated antiproliferative activity by reducing colony formation, highlighting its ability to impede cell survival and growth in vitro. Brassinin also exhibited anti-Warburg effects by suppressing glycolysis-related proteins like pyruvate kinase isoenzyme M2 (PKM2), glucose transporter 1 (Glut1), hexokinase 2 (HK2), and lactate dehydrogenase (LDH) in PC-3 cells, indicating an impact on the tumor’s energy metabolism. Its inhibitory effects on key proteins involved in promoting glycolysis, such as cellular myelocytomatosis oncogene (c-Myc), sirtuin 1 (SIRT1), and β-catenin, were noted, further emphasizing its potential as an anti-Warburg agent. Brassinin disrupted the interaction between SIRT1 and β-catenin, suggesting the involvement of c-Myc/SIRT1/β-catenin in its anti-Warburg effect. Moreover, brassinin triggered ROS production in PC-3 cells, significantly contributing to its antitumor effects. The use of the ROS scavenger N-acetyl-L-cysteine (NAC) reversed brassinin’s impact on the expressions of pro-PARP, pro-caspase 3, SIRT1, and β-catenin, highlighting the crucial role of ROS in brassinin’s antitumor properties [41]. Similar results were observed in the study by Hong et al. in 2021, where NAC effectively inhibited brassinin-induced ROS production in Huh7 cells (human hepatocellular carcinoma cells with mutated p53). It is interesting that this effect was not observed in Hep3B cells (human hepatocellular carcinoma cells, deleted P53) [17]. This variance in response to oxidant stimulus could be attributed to the differences in p53 phenotype and their respective reactions, as observed in studies on Huh7 and Hep3B cells [53,54]. Moreover, brassinin demonstrated regulatory effects on AKT and MAPK signaling pathways in both Huh7 and Hep3B cells. Additionally, the concurrent administration of brassinin with pharmaceutical inhibitors targeting JNK, ERK1/2, and P38 exhibited a more pronounced reduction in cell proliferation in both hepatocellular carcinoma cell lines compared to using the inhibitors alone [17].

Brassinin is identified as a potential antiangiogenic drug for future cancer treatment. Its potent antiangiogenic activity is attributed to its ability to stimulate Tie2 and FGFR1 degradation. Brassinin promotes the lysosomal degradation of Tie2 and both lysosomal and proteasomal degradation of FGFR1 in endothelial cells. This results in the down-regulation of the AKT and ERK pathways, ultimately inhibiting angiogenesis [42]. Tie2, a receptor tyrosine kinase primarily found in the endothelium, exhibits significant expression in the vascular system of tumors. Activation of Tie2 promotes endothelial cell survival, sprouting, migration, and the formation of capillary tubes [55]. Conversely, inhibiting Tie2 activity has demonstrated its ability to impede tumor angiogenesis and limit the growth of various cancer types, such as breast cancer, melanoma, and hepatocellular carcinoma, in numerous mouse models [56,57,58,59]. FGFR1, another receptor tyrosine kinase within the FGFR family, is also notably expressed in endothelial cells. Due to its role in tumor angiogenesis, FGFR1 is considered a promising target for therapeutic intervention. Interestingly, it has been observed that Tie2 and FGFR1 can physically interact with each other, leading to the phosphorylation of signal transducer and activator of transcription 3 (STAT3). The activation of STAT3 signaling is known to play a crucial role in regulating the formation of new blood vessels in various tumor types [60]. Considering the significant involvement of both Tie2 and FGFR1 in tumor angiogenesis, it is plausible to assume that the synergistic inhibition of these two pathways may contribute to the remarkable antiangiogenic efficacy of brassinin [42].

**Figure 4:**
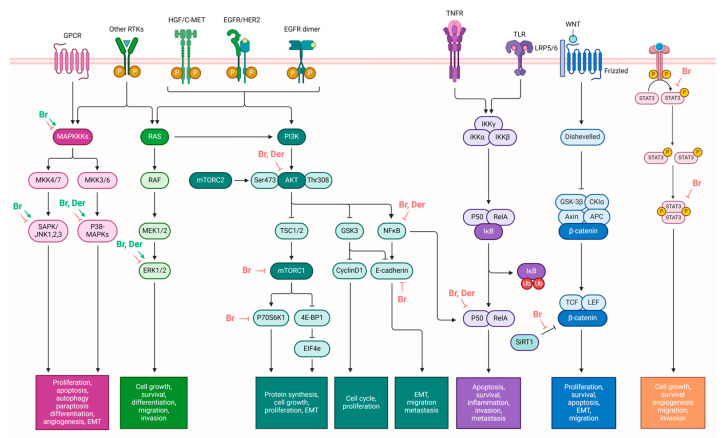
Indole phytoalexins as modulators of tumor signaling pathways. Green arrow—induction, red T bar—inhibition, Br—brassinin, Ca—camalexin, Der—derivatives. Adapted from “Signaling Pathways in Gastric Cancer” by BioRender.com (2024).

The research by Yang et al. in 2019 investigated the effects of brassinin on the epithelial–mesenchymal transition (EMT)-related cell signaling cascade. Their findings demonstrated that brassinin downregulates various mesenchymal markers while upregulating epithelial markers. It effectively suppresses the expression of TGF-β-induced fibronectin, vimentin, N-cadherin, the matrix metalloproteinases (MMP-9 and MMP-2), Twist, and Snail while increasing TGF-β-induced occludin and N-cadherin levels. Modulating these EMT-related factors leads to decreased proliferation and invasion in lung carcinoma cells, specifically A549 and H1299 cells. Brassinin also attenuates TGF-β-induced conversion into a spindle-shaped morphology and impedes cellular invasion and migration promoted by TGF-β. It can effectively inhibit the TGF-β-induced activation of oncogenic cascades, including those involving PI3K, Ras, and Rho GTPases. Furthermore, brassinin inhibits both constitutive and inducible phosphorylation of PI3K/Akt/mTOR/p70S6K/4E-BP1 in lung carcinoma cells [43]. The activation of the PI3K/Akt/mTOR/p70S6K/4E-BP1 pathway by TGF-β significantly contributes to encouraging cellular changes that support EMT, a crucial process in the advancement and spreading of cancer [61]. The insightful findings of this study contribute to the understanding of brassinin’s potential as a therapeutic agent in mitigating EMT-associated cell signaling cascade in lung carcinoma cells (Figure 4).

Brassinin has shown significant synergistic effects when combined with conventional chemotherapeutic agents. A study reported by Bakar-Ates and Ozkan in 2019 found that the combination of brassinin and imatinib exerted considerable anticancer effects by promoting the downregulation of MMP-9 mRNA expression in SW480 colon cancer cells [62]. In another study, Yang et al. (2021) discovered that combining brassinin therapy with a low dose of paclitaxel resulted in synergistic inhibition of cellular proliferation and promoted apoptosis in another type of colorectal cancer cells (HT-29 cells). By suppressing activation of the JAKs/STAT3 and PI3K/Akt/mTOR cascades, they demonstrated that brassinin enhanced the apoptotic and cytotoxic effects of paclitaxel [40]. Moreover, the combination of brassinin and doxorubicin, a drug used in colon cancer treatment, exhibited increased anticancer effects in HCT116 cells (human colorectal carcinoma). This combination prompted the activation of p53 and cleaved-PARP in HCT116 cells with the wild-type p53 gene [38].

### 3.2. Potential Impacts of Brassinin on Human Health

Brassinin has the potential to be employed as a depigmenting agent in medicinal or cosmetic products. Functioning as an inhibitor of melanin accumulation, brassinin achieves this by reducing the mRNA level of tyrosinase, which occurs through the inhibition of melanocyte-inducing transcription factor (MITF) translocation [35]. Tyrosinase serves as a critical enzyme in the initial stages of melanogenesis, while MITF acts as an essential transcription factor regulating tyrosinase [63,64].

Further research revealed brassinin’s effectiveness in inhibiting lipid accumulation during adipogenesis in a murine preadipocyte cell line (3T3-L1) and in suppressing obesity-induced inflammatory responses. This phytoalexin achieves inhibition of adipogenesis by modulating adipogenic factors differently. Specifically, it upregulates the expression of Kruppel-like factor 2 (KLF2), an anti-early adipogenic factor, and down-regulates CCAAT-enhancer-binding protein-β (C/EBPβ), an early adipogenic factor. Additionally, its inhibitory effect on obesity-induced inflammation operates through the activation of the nuclear factor erythroid 2-related factor–heme oxygenase-1 (Nrf2-HO-1) signaling pathway, suggesting its crucial role in the anti-inflammatory effects of brassinin [33]. Nrf2 and its target gene, HO-1, have been established as key players in obesity and insulin resistance etiology [65,66].

Brassinin may potentially reduce inflammatory processes through the suppression of monocyte-to-macrophage differentiation. It hampers this differentiation by reducing cell adhesion and the expression of differentiation markers C11β and CD36 in human monocytes. Additionally, it reduced the expression of pro-inflammatory cytokines or mediators in macrophages obtained from murine and human tissues, effectively suppressing LPS-induced inflammatory responses. The inhibitory effect of brassinin on the production of pro-inflammatory cytokines is associated with a reduction in nuclear translocation of Nu-clear factor kappa B (NF-κB) [34].

Results from the study by Han et al. (2017) suggest that brassinin could potentially exert an inhibitory effect on the inflammation of vascular endothelial cells [37]. In the context of vascular inflammation, oxidative stress plays a crucial role in initiating atherosclerosis, representing a vascular disorder [67]. The tumor necrosis factor alpha (TNF-α), identified as a critical mediator in the inflammatory pathway, is closely linked to the pathogenesis of numerous cardiovascular diseases, including atherosclerosis [68]. The remarkable effects of brassinin were observed, as pretreatment with brassinin significantly reduced TNF-α-induced intracellular reactive oxygen species (ROS) production in human umbilical vein endothelial cells (HUVECs). Furthermore, brassinin exhibited a significant dose-dependent inhibition of U937 monocyte cell adhesion to TNF-α-induced HUVECs. Treatment with brassinin also led to reduced expression levels of cell adhesion molecules, such as intercellular adhesion molecule-1 (ICAM-1), vascular cell adhesion molecule-1 (VCAM-1), and endothelial-selectin (E-selectin), following TNF-α stimulation in HUVECs. Moreover, pretreatment with brassinin demonstrated a reduction in the protein expression levels of nuclear factor NF-κB p65 in the nucleus, indicating a potential inhibition of NF-κB p65 nuclear translocation. The treatment with brassinin also substantially decreased the mRNA expression levels of interleukin-8 in a dose-dependent manner. Consequently, these findings suggest that brassinin holds promise as a potential therapeutic agent for the treatment of atherosclerosis [37].

Except for its anti-inflammatory effect, brassinin also exhibits antihyperglycemic, antioxidant, and protective effects on the liver, kidney, and pancreas during the onset of diabetes. The study by Xu et al. (2023) aimed to investigate the effects of brassinin in the treatment of experimentally induced diabetes in rats. After intraperitoneal injection of 35 mg/kg streptozotocin (STZ) to induce diabetes, rats were treated with 25 mg/kg of brassinin, using glibenclamide as a positive control. The study revealed that treatment with brassinin and glibenclamide significantly reduced glucose and HbA1c levels in diabetic rats while elevating insulin levels. Additionally, AST, ALP, and ALT levels in the experimental rats were measured, showing a significant increase in enzyme activities in diabetic rats and a substantial decrease in those administered with brassinin. The assessment of hepatic carbohydrate metabolic enzymes, including HK, F16BP, and G6P, indicated a considerable decrease in HK activity and a significant increase in G6P and F16BP activities in the diabetic liver. Brassinin enhanced glucose homeostasis and promoted glucose metabolism by increasing hepatic HK activity. Treatment with brassinin and glibenclamide prevented the reduction in antioxidant levels, indicating their restorative potential. Furthermore, the impact of brassinin on inflammatory markers in the pancreas, including IL-6, TNF-α, and IL-1β, was determined. Diabetic rats exhibited a substantial elevation in inflammatory markers, while treatment with brassinin and glibenclamide significantly reduced these marker levels, suggesting anti-inflammatory potential. The histological examination of the pancreas, kidney, and liver revealed protective effects of brassinin against STZ-induced damage, supporting its potential therapeutic role in mitigating diabetes-related complications) [36]. The antidiabetic properties of brassinin are attributed to the presence of dithiocarbamate ester in its composition. This component has been demonstrated to have antihyperglycemic properties in previous studies [36,69,70].

## 4. Camalexin

Camalexin, initially discovered in *Camelina sativa* leaves infected with *Alternaria brassicae*, functions as the primary indole phytoalexin in *Arabidopsis thaliana* and other cruciferous plants [71]. Its role in plant defense has been extensively studied, revealing its involvement in chemical defense mechanisms. Moreover, research has demonstrated its cytotoxicity against *Trypanosoma cruzi*, a human protozoan pathogen [6]. 

### 4.1. Anticancer Potential of Camalexin

Camalexin has demonstrated its ability to inhibit the growth of different cancer cell lines [14,16,72,73]. Nonetheless, the precise mechanism through which camalexin inhibits cancer cell proliferation remains unresolved.

The anticancer potential of camalexin is evident in its ability to enhance the expression of the Aryl hydrocarbon receptor (AhR) target genes, facilitate the nuclear translocation of AhR, and activate AhR-mediated transcription. Additionally, camalexin inhibited mammosphere formation in AhR-expressing breast cancer cells more than in the breast cancer cells that lacked AhR expression [74]. AhR is implicated in both breast cancer progression and drug resistance [75,76]. Studies indicate that AhR promotes breast cancer malignancy. However, it has also been observed that certain AhR agonists or activators can suppress various types of cancer. Therefore, AhR is believed to have a dual function in cancer [77,78]. The results of the study by Yamashita et al. (2022) indicate that the inhibition of cell proliferation, migration, and mammosphere formation by camalexin specifically depends on the activation of AhR in ER-negative, PgR-positive, and HER2-negative breast cancer cell lines, such as MCF-7 and T47D [74].

Camalexin also exhibited toxicity towards human myeloid leukemia cell lines (HL60 and NB4) with minimal toxicity to normal human peripheral blood mononuclear cells. It induced apoptosis (Figure 3) in those leukemia cells via the activation of caspase-3 and caspase-9 [16], consistent with previous findings in the human T lymphocyte (Jurkat) cell line [72]. After exposure to camalexin, ER stress markers such as the phosphorylated protein kinase R-like endoplasmic reticulum kinase (phos-PERK), the phosphorylated eukaryotic initiation factor 2 alpha (phos-eIF2α), ATF4 and CHOP were significantly upregulated in the aforementioned leukemia cells. ER stress contributed to camalexin-induced apoptosis, as evidenced by the observed inhibition of apoptosis when an ER stress inhibitor and siRNA against PERK (Protein kinase R-like endoplasmic reticulum kinase) were applied. Camalexin also reduced Mcl-1 in leukemia cells. Mcl-1 is crucial in regulating the intrinsic pathway of apoptosis and is recognized for its role in promoting cell survival by inhibiting apoptosis. Additionally, following camalexin treatment, there was a notable increase in ROS levels, SOD activity, CAT activity, and GSSG levels, accompanied by a decrease in GSH levels. In xenograft models, camalexin effectively suppresses tumor growth in NB4 and HL-60 without significant side effects. Camalexin shows promising anti-leukemia activity by activating caspases, inducing ER stress, and generating ROS [16]. Notably, camalexin was found unable to induce ROS in erythrocytes in a recent study. This discrepancy might arise from variations in cell types. The author hypothesizes that camalexin could potentially trigger ROS in cancer cells while sparing normal cells [79]. 

Camalexin and brassinin synergistically targeted colorectal cancer cells (Caco-2) due to their multifaceted mechanisms and amplified cytotoxic effects. Both phytoalexins were cytotoxic to Caco-2 cells, with camalexin being more potent. The combination of brassinin and camalexin was the most effective against cancer cells (Caco-2), while non-cancer cells (CCD-Co18) were less sensitive to these compounds. The IC50 values for Caco-2 cells, when these phytoalexins were used alone, were relatively high, with 264.06 μM for brassinin and 139.27 μM for camalexin after 48 h. The mixture of brassinin and camalexin was more potent than each compound alone (IC50 85.81 μM). Both phytoalexins induced oxidative stress in cancer cells, leading to apoptosis and, at higher concentrations, necrosis. In addition, signs of pyroptosis were observed in cells exposed to camalexin, as indicated by the increased activity of caspase 1 [80]. This caspase serves as the primary effector protease, playing a key role in initiating the pyroptotic cell death pathway [81]. The simultaneous initiation of other types of cell death, such as pyroptosis, is intriguing, given that typical chemotherapy relies on inducing apoptosis in cancer cells, and chemoresistance involves evading apoptosis [80]. 

The overview of the potential anticancer effects of brassinin and camalexin are summarized in Table 1.

### 4.2. Potential Impacts of Camalexin on Human Health

The study conducted by Manasa and Chitra in 2020 suggested that camalexin possesses neuroprotective properties, which could be potentially beneficial in the context of Parkinson’s disease. The assessment of camalexin’s protective and antioxidant activity involved various assays, including ABTS, DPPH, and FRAP, revealing its effectiveness in scavenging free radicals in a concentration-dependent manner. These findings confirm the potential of camalexin in reducing oxidative stress-related damage, especially in the context of Parkinson’s disease [82]. 

In addition to its role in defense, camalexin exhibits intriguing effects on erythrocytes. It stimulates eryptosis, a process involving cell shrinkage and membrane scrambling with phosphatidylserine translocation on the erythrocyte surface. This effect is associated with increased cytosolic Ca^2+^ activity and requires Ca^2+^ entry from the extracellular space. Camalexin-induced cell membrane scrambling also involves protein kinase C and caspase activation. Notably, camalexin induces these effects without increasing ceramide levels or inducing oxidative stress [79], which are commonly associated with eryptosis triggers [83]. The potential clinical implications of camalexin use warrant careful consideration. While it demonstrates neuroprotective and antioxidant properties, its eryptotic effects on erythrocytes could lead to side effects such as anemia and an elevated risk of thrombosis if effective concentrations are achieved in patients’ plasma [79]. This complex interplay of beneficial and potentially adverse effects underscores the need for a thorough investigation before considering camalexin for therapeutic applications.

## 5. Antiproliferative Effects of Synthetic Analogues of Indole Phytoalexins

While natural compounds are a significant source of clinically used anticancer drugs, their properties, such as lower therapeutic efficacy, unfavorable pharmacokinetic characteristics, or unexpected toxicity, sometimes limit their direct use in therapeutic practice. Consequently, many drugs based on natural products are either derivatives of natural products or synthetic molecules inspired by the structure of natural products [84]. Another limitation of their direct use is the low yield from fresh plant tissues (for example, in the case of indole phytoalexins, it is 1–5 mg per 1 kg of fresh tissue from cruciferous plants) [26]. Synthetic analogs of natural compounds can offer several advantages over their parent compounds in cancer therapy. First, synthetic analogs can be designed to exhibit potent anticancer activity and selectivity to cancer cells, resulting in increased therapeutic efficacy and reduced risk of side effects commonly associated with traditional chemotherapy. Second, synthetic analogs can have optimized pharmacokinetic properties, which can result in better drug delivery to the tumor site and increased efficacy of the treatment. Third, through synthetic chemistry, it is possible to introduce structural modifications to parent natural compounds, allowing for the fine-tuning of their properties. These modifications may improve solubility, stability, cellular uptake, and target specificity, enhancing their therapeutic potential. These reasons prompted us to include a chapter on synthetic analogs of indole phytoalexins, as we have been intensively researching this area over the past two decades.

The majority of indole phytoalexins and their synthetic derivatives exhibit chirality [26,85]. Studies have demonstrated that chemically modifying the structure of indole phytoalexins results in increased antiproliferative activities compared to the original natural compounds [15,86,87,88,89,90]. 

In recent years, we have undertaken the synthesis and testing of numerous novel derivatives of indole phytoalexins. A study conducted by Tischlerova et al. (2017) aimed to investigate the antiproliferative and pro-apoptotic effect of newly synthesized 2′-aminoanalogues of 1-methoxyspirobrassinol methyl ether on colorectal carcinoma cells (HCT116). Among the tested compounds, (±)-trans-1,2-dimethoxy-2′-(3,5-bis-trifluoromethylphenylamino)spiro{indoline-3,5′ [4′,5′]dihydrothiazol} (substance K-453) exhibited the highest antiproliferative effect on HCT-116 cancer cells (32,2 μM). We found that K-453 suppressed the growth of HCT116 cells and induced apoptosis, as confirmed by various assays. Furthermore, K-453 caused an increase in the G0/G1 phase of the cell cycle and a corresponding increase in cells with sub-G1 DNA content, indicative of programmed cell death. The number of apoptotic HCT116 cells significantly increased over time, as demonstrated by annexin V/PI, AO/PI staining, and PARP cleavage. Mitochondrial dysfunction, a key aspect of apoptotic cell death, was observed following K-453 treatment, leading to the loss of mitochondrial membrane potential and the release of cytochrome c into the cytosol with subsequent caspase-9 and caspase-3 activation. Moreover, K-453 also influenced the Bcl-2 family proteins, with an increase in Bcl-2 phosphorylation and BAD dephosphorylation. Additionally, the study examined the influence of K-453 on several signaling pathways, including p38 MAPK, ERK1/2, Akt, and NF-κB. Phosphorylation of p38 MAPK, linked to Bcl-2 inactivation, was observed, whereas phosphorylation of Akt and ERK1/2 decreased post-K-453 treatment. Lower levels of NF-κB/p65 and p50 were detected, indicating a disruption in the NF-κB pathway. In summary, K-453 induced caspase-dependent apoptosis in colon cancer cells through the mitochondrial pathway. Furthermore, it activated the p38 MAPK pathway while deactivating the ERK1/2, Akt, and NF-κB signaling pathways (Figure 4). These significant findings underscore the potential of spiro-indole derivatives as apoptotic agents in colon cancer treatment [89].

Within our research, we prepared new biologically active trifluoromethyl (CF3) containing indole derivatives. CF3 functional group holds significant importance in the field of pharmaceutical chemistry and is a component of drugs such as fluoxetine, celecoxib, and efavirenz. The antiproliferative activity of the new fluorinated 2,2′-diaminoanalogues of 1-methoxyspirobrassinol methyl ether was tested on multiple cell lines, including Jurkat, CEM (acute T-lymphoblastic leukemia); MCF-7, MDA-MB-231 (triple negative breast cancer); A-549, HeLa (cervical adenocarcinoma); HCT116, CaCo-2; and a non-malignant cell line NIH 3T3 (murine fibroblasts). The most effective was trans-(±)-1-Methoxy-2-[3,5-bis(trifluoromethyl)phenylamino]-2′-[3,5-bis(trifluoromethyl)phenylamino]spiro{indoline-3,5′-[4′,5′]dihydrothiazole} on CEM cell line (IC50 = 1 μM). This analog was even more effective than cisplatin, which is used as a reference antitumor compound. Additionally, the newly synthesized derivatives also exhibited certain selectivity with a lower antiproliferative effect on non-tumor cell lines compared to tumor cell lines [90].

Our researchers successfully synthesized the new cruciferous phytoalexin (*S*)-(−)-spirobrassinin and its (±)-2′-amino analogs using a convenient method. These newly synthesized compounds underwent in vitro screening to evaluate their antiproliferative/cytotoxic effects on six human cancer cell lines: Jurkat and CEM, MCF-7 and MDA-MB-231, HeLa, and A-549 using the MTT assay. Among the tested compounds, ((±)-2′-[4-(Trifluoromethyl)phenylamino]spiro[indoline-3,5′-[4′,5′]dihydrothiazole]-2-one) showed the highest activity against Jurkat cells (IC50 = 26.1 µM). Additionally, it demonstrated notably lower toxicity compared to cisplatin on HUVEC cells. These results indicate the importance of the CF3 group for enhanced biological activity [91].

Furthermore, novel 5-bromo derivatives of indole phytoalexins were tested for their antiproliferative/cytotoxic effects on human cancer cell lines Jurkat, MCF-7, MDA-MB-231, A-549, HeLa, HCT116, and CaCo-2 and a non-malignant cell line NIH 3T3. The strongest inhibitory effect was exhibited by *N*-[[5-bromo-1-(tert-butoxycarbonyl)indol-3-yl]methyl]-*N*’-[4-(trifluoromethyl)phenyl]thiourea on Jurkat cells with an IC50 of 5.1 µM. This analog also demonstrated lower toxicity on 3T3 cells compared to cisplatin [92].

The study by Budovská et al. (2021) focuses on the design and synthesis of new 2′-aminoanalogues of 5-fluorospirobrassinin. The design strategy involves replacing a hydrogen atom with a fluorine atom at the C-5 position of indole phytoalexins. The synthesized compounds were tested for their cytotoxic activity against various human cancer cell lines as well as noncancerous cells. Among the tested compounds, (±)-5-Fluoro-2′-(3,4-dichlorophenylamino)spiro{indoline-3,5′-[4′,5′]dihydrothiazole}-2-one exhibited the highest effectiveness against the cancer cell lines, particularly HCT116 and Jurkat (IC50 values 30.7 μM and 29.1 μM), while showing no cytotoxicity towards noncancerous HUVEC cells. However, overall, the novel 5-fluoro analogs did not demonstrate improved anticancer activity compared to the lead compounds. The presence and position of a fluorine atom in the indole portion of the molecules were not found to be critical factors in achieving anticancer activity, based on the preliminary structure–activity relationship study [93].

**Figure 5:**
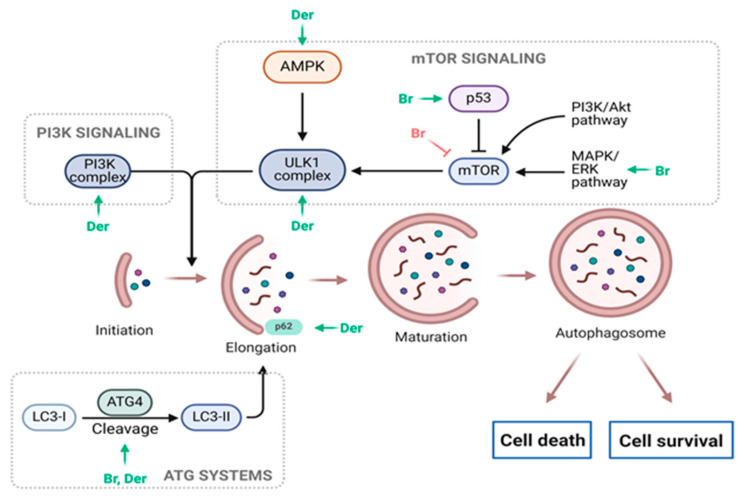
Indole phytoalexins as modulators of autophagy induction. Green arrow—induction, red T bar—inhibition, Br—brassinin, Ca—camalexin, Der—derivatives. Adapted from “Autophagy in Cancer Pathways”, by BioRender.com (2024).

Moreover, a new series of bis-indole analogs incorporating a phenyl linker derived from the indole phytoalexin 1-methoxyspirobrassinol methyl ether were synthesized and designed. Remarkably, substance 49 (*N*,*N*′-(1,4-phenylene)bis{*N*′-[1-(tert-butoxycarbonyl)indol-3-yl]methyl (urea)}) exhibited significant growth inhibition in A549 cells (8.7 µM) while displaying minimal toxicity towards noncancerous cells (MCF10A and Cos-7). Surprisingly, investigations into its mechanism of action revealed that substance 49 did not affect the cell cycle or induce apoptosis. However, subsequent analyses indicated that substance 49 induced autophagy, as evidenced by increased phosphorylation or expression of various autophagy markers such as Beclin-1, AMPK, ULK1, p62, Atg7, and LC3A/B (Figure 5). Moreover, when combined with chloroquine, an autophagy inhibitor, substance 49 led to cell cycle arrest at the G1 phase and an elevated number of cells in late-stage apoptosis. Based on these findings, it is proposed that autophagy likely serves a defensive role and is not directly involved in the antiproliferative effects of substance 49. Furthermore, substance 49 was found to deplete GSH (glutathione) in cancer cells, increasing their sensitivity to cancer treatments. This was demonstrated by the potentiation of cisplatin cytotoxicity in indole phytoalexin-treated cells. These results provide intriguing and novel insights, suggesting that substance 49-induced GSH depletion could be utilized to enhance the effectiveness of anticancer drugs [94]. 

In our recent study, Zigová et al. (2024), the significant antiproliferative properties of 1-methoxyisobrassinin, a regioisomer of the natural indole phytoalexin 1-methoxybrassinin, were demonstrated in both sensitive and cisplatin-resistant ovarian cancer cells (A2780) in vitro (IC50 3.62 µM and 7 µM, respectively). 1-methoxyisobrassinin induced ROS production in both cell lines, associated with apoptotic cell death machinery induction. ROS-induced DNA damage caused alterations in the cell cycle distribution, particularly affecting the S and G2/M phases. These changes were accompanied by modifications in key cell cycle regulatory proteins, such as proliferating cell nuclear antigens (PCNA). Additionally, there was activation of caspase-9, PARP cleavage, and mitochondrial dysfunction. The role of ROS in the antiproliferative effect of 1-methoxyisobrassinin was confirmed by experiments with NAC, which significantly suppressed ROS production and various apoptosis-related processes. Moreover, modulation of protein expression/phosphorylation, such as LC3A/B, ULK1, or phosphatase and tensin homolog (PTEN), in resistant A2780cis cells suggested possible autophagy activation (Figure 5). The activation of autophagy could represent an attempt by resistant tumor cells to survive [95].

At our institution, we conducted an evaluation of amino analogs of 1-methoxyisobrassinin for their antiproliferative activities. Among them, *N*-[(1-methoxyindol-2-yl)methyl]-*N*′-[3,5-bis(trifluoromethyl)phenyl]thiourea, bearing two CF3 groups on the benzene ring, displayed the highest potency. It exhibited significant antiproliferative activities against Jurkat (IC50 = 7.6 μM) and MCF-7 cell lines (IC50 = 8.7 μM) in comparison to cisplatin (IC50 = 16.2 μM and 15.6 μM, respectively). Moreover, this particular compound showcased greater activity against cancer cells than non-cancer cells (IC50 = 26.7 μM in the murine fibroblast cell line NiH 3T3). Furthermore, this study specifically investigated the antiproliferative effect of erucalexin, which stands out among natural indole phytoalexins due to its unique C-2 carbon substituent, while other indole phytoalexins have carbon substituents at C-3. However, erucalexin generally exhibited poor antiproliferative effects on cancer cell lines. The screening of amino analogs of erucalexin revealed that they demonstrated good activity only against the Jurkat cell line. Comparatively, the (±)-erucalexin derivatives were less active than their parent regioisomers, except in the case of Jurkat cells. The findings of this study suggest that the replacement of the methylsulfanyl group with a phenylamino group in the structure of erucalexin results in an increase in antiproliferative activity [96].

The study by Csomos et al. (2017) investigated the growth inhibition effects of isobrassinin and isocyclobrassinin analogs containing an indole-2-carboxamide moiety on a panel of human cell lines, including HeLa, A431 (human squamous carcinoma), A2780, and MCF7. The cyclic analogs, characterized as ring-closed 4-arylimino-thiazines, demonstrated moderate inhibition of cell growth (up to 67.2% at 10 μM on the A431 cell line). Notably, indole analogs containing benzothiazole ring with methoxy groups also exhibited a relatively good inhibitory effect on the A2780 cell line (60.4% at 10 μM). The analog of isocyclobrassinin with the 1,3-thiazino[5,6-b]indol-4-one skeleton exhibited the highest antiproliferative activity (89.6% at 10 μM) on the A2780 cell line. The results indicated that this analog displayed growth inhibition comparable to that of cisplatin (83.6% at 10 μM) [97].

Within our research facility, we investigated the sensitizing effect of thiazine[6,5-b]indole, both alone and in combination with the heat shock protein (Hsp) 90 inhibitor 17-dimethylaminoethylamino-17-demethoxygeldanamycin (17-DMAG), on cisplatin-resistant ovarian cancer cells (A2780cis). 17-DMAG alone exhibited a weak impact on cell proliferation in A2780cis cells. However, its combination with thiazine[6,5-b]indole (2-(4′-fluorphenylamino)-4*H*-1,3-tiazino[6,5-b]indol) significantly enhanced the antiproliferative activity, reducing the IC50 from 30 µM to 8.6 µM. Additionally, this combination increased pro-apoptotic activity of 17-DMAG [98]. Numerous studies have highlighted the substantial involvement of the Hsp90 protein in contributing to cisplatin resistance in cancer cells. It has been established that Hsp90 inhibitors can effectively counteract resistance or amplify the sensitivity of cancer cells [99,100]. Particularly noteworthy is the discovery that combining Hsp90 inhibitors with conventional treatments or natural compounds further enhances their efficacy [100,101].

The chemical structures of the most effective synthetic analogs of indole phytoalexins tested at our institution, as well as their antiproliferative activity, are shown in Table 2.

## 6. Conclusions

Indole phytoalexins exhibit a diverse range of potential pharmacological properties, including antibacterial, antifungal, antiobesity, anti-inflammatory, antimelanogenic, antidiabetic, antiatherosclerotic, neuroprotective, and notably, anticancer effects. Brassinin, a prominent phytoalexin, has been found to induce apoptosis, autophagy, and paraptosis through the activation of the MAPK signaling pathway. Its ability to trigger ROS production significantly contributes to its antitumor effects. Furthermore, brassinin exhibits anti-Warburg effects by suppressing glycolysis-related proteins. This indole phytoalexin demonstrates antiangiogenic activity by stimulating the degradation of Tie2 and FGFR1. Additionally, brassinin modulates the epithelial–mesenchymal transition and inhibits TGF-β-induced oncogenic cascades, offering promising opportunities for carcinoma treatment. When combined with conventional chemotherapeutic agents, brassinin demonstrates significant synergistic effects, enhancing therapeutic efficacy against cancer. Similarly, camalexin displays anticancer potential through AhR activation, ER stress induction, and ROS generation, with promising outcomes observed in combination therapy. Its neuroprotective and antioxidant properties suggest potential applications in mitigating oxidative stress-related damage, although careful consideration is needed due to its eryptotic effects on erythrocytes. Chemical modifications of indole phytoalexins have been successful in enhancing their antiproliferative activities. Some derivatives show potency comparable to conventional chemotherapy drugs. Additionally, the selectivity of these derivatives toward cancer cells offers the potential for reducing systemic toxicity. Further research is warranted to elucidate their full therapeutic potential. 

## Figures and Tables

**Table 1:** The anticancer potential of brassinin and camalexin (update since 2016). Arrows indicate an increase (↑) or decrease (↓) in the levels/activity of the molecules.

**Brassinin**	**Anticancer Potential**	**Molecular Target**	**Relevant Reference(s)**
	The modulation of pathways involved in cell survival, proliferation, and growth	↓ JAK-STAT3 pathway ↑ MAPK pathway ↓ PI3K/Akt/mTOR pathway	[39,40,43]
	Induction of cancer cell death	↑ p53, ↑ p21 ↑ caspase-8, caspase-9, and caspase-3 ↑ PARP cleavage ↑ Sub-G1 phase distribution in cells, ↑ G0/G1 ratio, ↓ S, G2/M ↑ Bax expression, ↓ Bcl-2 expression ↑ Phosphorylation of JNK, ERK, and p38 ↑ ROS production ↑ Mitochondrial damage ↑ ER stress-related proteins (ATF4 and CHOP) ↑ Cytoplasmic vacuolation ↑ GSH/GSSG ratio disruption ↓ Expression of CNOT2 ↑ Expression of LC3 ↓ Alix expression	[17,38,39,41,43,44]
	Anti-Warburg effect	↓ PK2, ↓ Glut1, ↓HK2, ↓LDH, ↓c-Myc, ↓SIRT1, ↓ β-catenin	[41]
	Inhibition of angiogenesis	↓ Tie2, ↓ FGFR1	[42]
	EMT modulation	↓ TGF-β-induced fibronectin, ↓ vimentin, ↓ N-cadherin, ↓ MMP-9, ↓ MMP-2, ↓Twist, ↓ Snail, ↑ TGF-β-induced occludin, ↑ N-cadherin	[43,62]
**Camalexin**	Modulation of AhR Pathway	↑ Expression of AhR target genes ↑ Nuclear translocation of AhR ↑ AhR-mediated transcription ↓ Mammosphere formation in AhR-expressing breast cancer cells	[74]
	Induction of cancer cell death	↑ Caspase-3 and caspase-9 activity ↑ ER stress markers (phos-PERK, phos-eIF2α, ATF4, CHOP) ↓ Mcl-1 levels ↑ ROS levels ↑ SOD and CAT activity ↑ GSSG levels, ↓ GSH levels ↑ caspase 1 (pyroptosis)	[16,80]
	Suppression of tumor growth in NB4 and HL-60 tumor xenograft models	↓ tumor volume, ↑ caspase-3 and caspase-9	[16]

**Table 2:** Antiproliferative effects of synthetic analogs of indole phytoalexins.

Group of Analogs	The Most Effective Analog	Cancer Cell Line	IC_50_ (μM)	Reference
2′-aminoanalogues of 1-methoxyspirobrassinol methyl etether	(±)-*trans*-1,2-dimethoxy-2′-(3,5-bis-trifluoromethylphenylamino)spiro{indoline-3,5′ [4′,5′]dihydrothiazol} 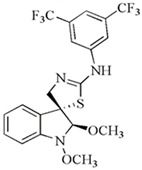	HCT-116	32.2	[89]
2′-aminoanalogues of spirobrassinin	(±)-2′-[4-(Trifluoromethyl)phenylamino]spiro[indoline-3,5′-[4′,5′]dihydrothiazole]-2-one 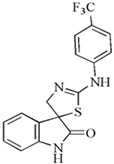	Jurkat	26.1	[91]
2′-aminoanalogues of cyclobrassinin	2-(4′-fluorphenylamino)-4*H*-1,3-tiazino[6,5-b]indol 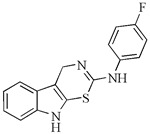	A2780cis	30	[98]
2′-aminoanalogues of 5-fluorospirobrassinin	(±)-5-Fluoro-2′-(3,4-dichlorophenylamino)spiro{indoline-3,5′-[4′,5′]dihydrothiazole}-2-one 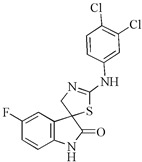	Jurkat	29.1	[93]
2,2′-diaminoanalogues of 1-methoxyspirobrassinol methyl ether	*trans*-(±)-1-Methoxy-2-[3,5-bis(trifluoromethyl)phenylamino]-2′-[3,5-bis(trifluoromethyl)phenylamino]spiro{indoline-3,5′-[4′,5′]dihydrothiazole} 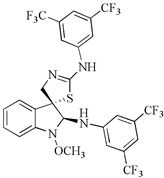	CEM	1	[90]
Aminoanalogues of 1-methoxyisobrassinin	*N*-[(1-methoxyindol-2-yl)methyl]-*N*′-[3,5-bis(trifluoromethyl)phenyl]thiourea 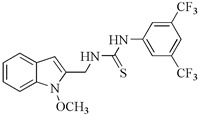	Jurkat	7.6	[96]
Aminoanalogues of 5-bromo-1-Boc-brassinin	*N*-[[5-Bromo-1-(tert-butoxycarbonyl)indol-3-yl]methyl]-*N*’-[4-(trifluoromethyl)phenyl]thiourea 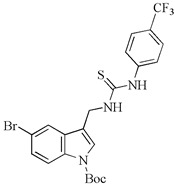	Jurkat	5.1	[92]
Bis-indole urea analogues with a phenyl linker	*N*,*N*′-(1,4-phenylene)bis{*N*′-[1-(tert-butoxycarbonyl)indol-3-yl]methyl (urea)} 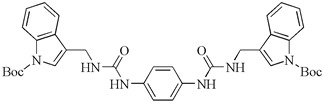	A549	8.7	[94]
Regioisomer of 1-methoxybrassinin	1-methoxyisobrassinin 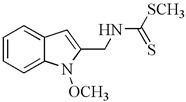	A2780 A2780cis	3.62 7.00	[95]

## Data Availability

Not applicable.
